# Widespread persistent changes to temperature extremes occurred earlier than predicted

**DOI:** 10.1038/s41598-018-19288-z

**Published:** 2018-01-17

**Authors:** Chao Li, Yuanyuan Fang, Ken Caldeira, Xuebin Zhang, Noah S. Diffenbaugh, Anna M. Michalak

**Affiliations:** 1 Department of Global Ecology, Carnegie Institution for Science, Stanford, California, USA; 2 Pacific Climate Impacts Consortium, University of Victoria, Victoria, British Columbia Canada; 3 Climate Research Division, Environment and Climate Change Canada, Toronto, Ontario Canada; 4 Department of Earth System Science, Stanford University, Stanford, California, USA; 5 Woods Institute for the Environment, Stanford University, Stanford, California, USA

## Abstract

A critical question for climate mitigation and adaptation is to understand when and where the signal of changes to climate extremes have persistently emerged or will emerge from the background noise of climate variability. Here we show observational evidence that such persistent changes to temperature extremes have already occurred over large parts of the Earth. We further show that climate models forced with natural and anthropogenic historical forcings underestimate these changes. In particular, persistent changes have emerged in observations earlier and over a larger spatial extent than predicted by models. The delayed emergence in the models is linked to a combination of simulated change (‘signal’) that is weaker than observed, and simulated variability (‘noise’) that is greater than observed. Over regions where persistent changes had not occurred by the year 2000, we find that most of the observed signal-to-noise ratios lie within the 16–84% range of those simulated. Examination of simulations with and without anthropogenic forcings provides evidence that the observed changes are more likely to be anthropogenic than nature in origin. Our findings suggest that further changes to temperature extremes over parts of the Earth are likely to occur earlier than projected by the current climate models.

## Introduction

Earlier work has investigated the time of emergence (TOE) of future persistent changes to a variety of climate variables^1–5^, including mean temperature^6,7^, mean precipitation^8,9^, regional sea-level^10^, Greenland Ice Sheet mass^11^, and temperature extremes^2–5^. Based on historical observations and climate model simulations, a few studies have also documented persistent changes to seasonal-scale mean temperatures^12^ and seasonal-scale extreme temperatures^2,4^ that have already occurred. However, due to the paucity of homogeneous long-term records of daily temperature, observational evidence supporting the emergence of persistent changes to daily-scale extreme temperatures is limited to a few small regions (e.g., Europe and Central England)^2,4^.

Here we use the HadEX2^13^ observational dataset for 1901–2005 to assess whether persistent changes in the distribution of daily-scale extreme temperatures have already occurred. We then use a suite of climate model simulations from the Coupled Model Intercomparison Project (CMIP5) to assess the likelihood of the observed changes with and without anthropogenic forcings. The CMIP5 ensemble includes simulations with historical natural and anthropogenic forcings (‘Historical’), historical greenhouse gas forcing (‘HistoricalGHG’), and historical natural forcing only (‘HistoricalNat’), as well as long pre-industrial control simulations (‘piControl’)^14^ (see Table S1 for the used climate models and simulations).

We analyze four extreme temperature indices, defined by the Expert Team on Climate Change Detection and Indices (“ETCCDI”)^15^: the percentages of extreme hot nights (TN90p), extreme hot days (TX90p), extreme cold nights (TN10p) and extreme cold days (TX10p) per year (see Table S2 for definitions). Compared to ‘extreme extremes’ (e.g., the hottest day of a year), the percentile-based indices are less affected by internal variability^16^, and thus offer a robust way to analyze the emergence of persistent changes while still representing important indicators for impacts on human and natural systems. For example, an increase in the number of unusually hot days and nights would have direct consequences for public health, agriculture, and energy demand^17,18^.

To detect the emergence of persistent changes in the distribution of a given extreme index, we compare the statistical distribution during a 1921–1950 baseline period to distributions during subsequent 30-year periods, using a moving window approach with a 1-year time step. A persistent change in the distribution is considered to have emerged if and only if the distributions for a given period (e.g., 1955–1984) and all subsequent periods (e.g., 1956–1985, 1957–1986, …, 1976–2005) are different from the baseline distribution, based on a two-sample Kolmogorov-Smirnov (K-S) test at 5% significance^6,9,12^. The last year of the initial emergence window is taken as the TOE (e.g., 1984, if 1955–1984 is the earliest time window after which there is persistent emergence). Because a change appearing close to the end of the period of record might represent a temporary emergence^2,3,19^, we specify that the change in the distribution must have been persistently evident by the year 2000 or earlier. Although this is not enough to completely rule out a temporary emergence, this limitation is necessary in the present study given the limited length of the available observational record. Because the observed global mean surface temperature over the period 1901–1920 was anomalously cold^20^, we exclude this period in order to avoid biasing the analysis towards artificially early detection of emergence. We note that our key findings are robust to moderate changes in baseline period and moving window width (Fig. S1).

## Results and Discussion

**Figure 1:**
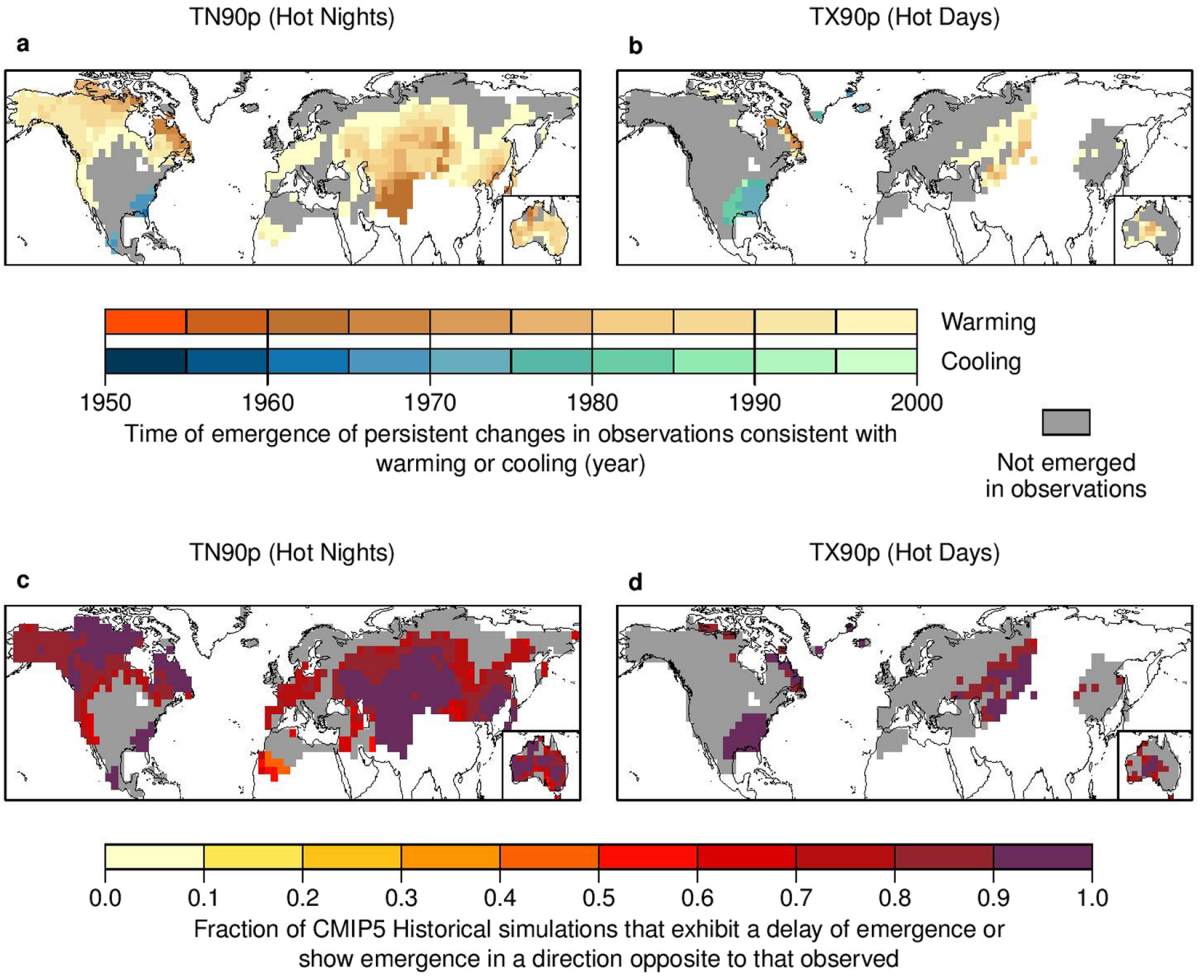
Persistent changes to TN90p (hot nights) and TX90p (hot days) have already occurred over large parts of the Earth and climate models underestimate these persistent changes. Top panels show time of emergence (TOE) of persistent changes to TN90p (**a**) and TX90p (**b**) derived from HadEX2 observations. Warm (cool) color marks regions where the emergence of persistent changes occurs in the direction consistent with warming (cooling). Gray color marks regions for which there is no emergence in HadEX2 observations by the year 2000. White regions have no data. See Fig. S7 for CMIP5 results corresponding to these panels. Bottom panels show the fraction of CMIP5 ‘Historical’ simulations that exhibit a delay of emergence of persistent changes to TN90p (**c**) and TX90p (**d**) or show emergence in a direction opposite to that observed (see Methods). It is noted that simulated emergence in the opposite direction to observed is restricted mainly to the ‘warming hole’ in southeast/central USA and to a few ensemble members (see Fig. S8). See Fig. S2 for TN10p (cold nights) and TX10p (cold days). The map is produced using R version 3.0.3 software (https://www.r-project.org/).

We find that some persistent changes in the distribution of temperature extremes occurred as early as the 1960s (i.e., about one and half decades from the baseline period) (Fig. 1a,b for TN90p and TX90p and Fig. S2a,b for TN10p and TX10p). By the year 2000, persistent changes to the distributions of TN90p and TN10p had occurred over the majority of land covered by the HadEX2 observations (65% for TN90p and 70% for TN10p), while changes in the distributions of TX90p and TX10p had occurred for a substantial fraction (22% for TX90p and 32% for TX10p). The TOE was as early as the 1960s for nighttime temperature extremes in some regions, including parts of Eurasia, the Asia-Pacific region, and Australia. We obtain very similar results using a 5-year block-bootstrap K-S test, which minimizes the influence of autocorrelation of temperature extremes on the original K-S test (Fig. S3; see the description for the block-bootstrap K-S test in the figure caption). Considering the relatively higher signal-to-noise ratio for temperature extremes in the tropics where these is no observational data, we suspect that persistent changes in the distribution of temperature extremes may have had occurred in the tropics as well. Further, we find that persistent changes to temperature extremes, especially the nighttime temperature extremes, tend to emerge earlier and are more widespread than persistent changes to annual mean temperature (Fig. S4). This is probably because a small shift in the distribution of daily temperature may substantially affect the occurrence probability of extremes hot/cold days and nights^21^. The use of different observational datasets for deriving the percentile-based extreme temperature indices and for annual mean temperature might also play a role.

**Figure 2:**
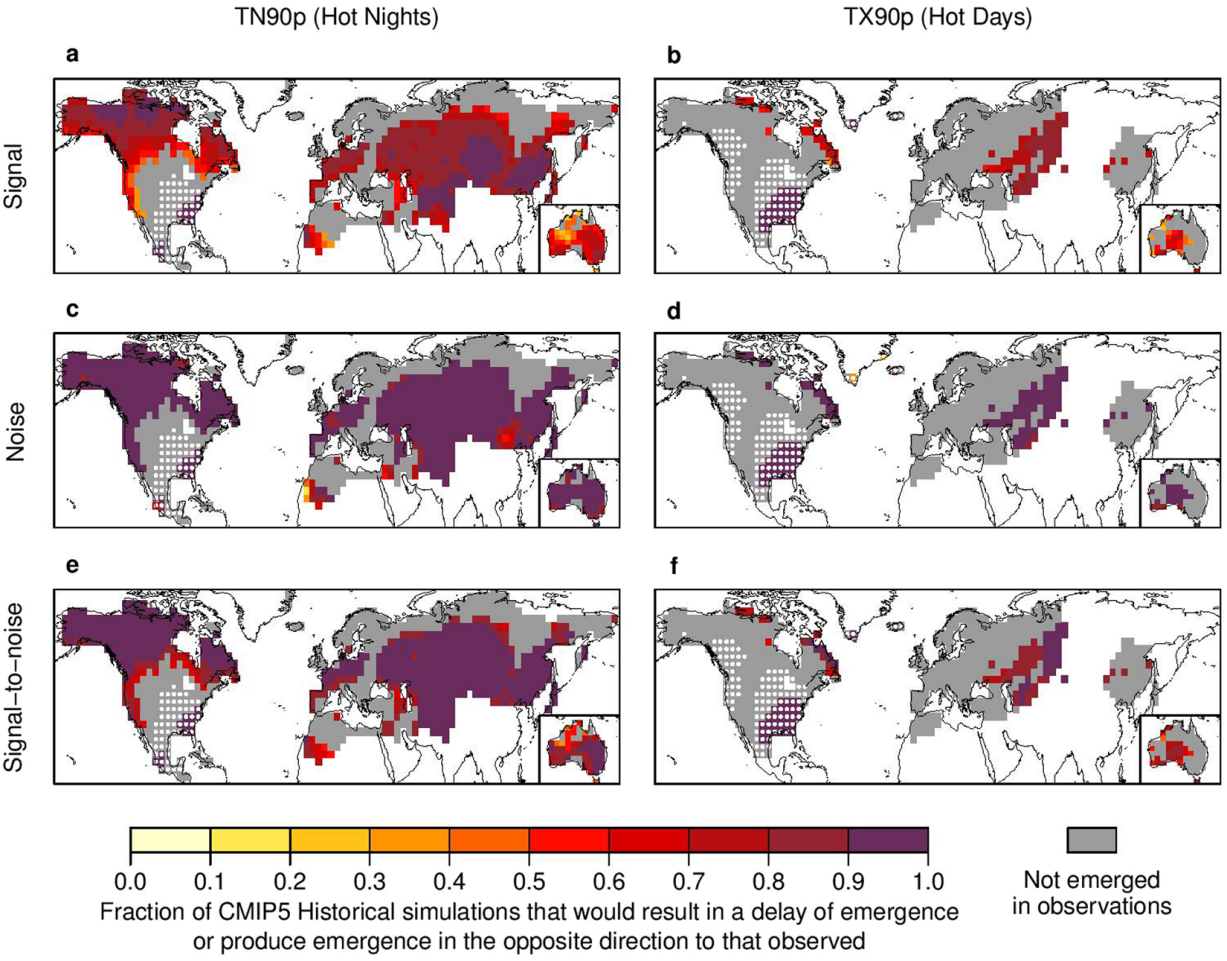
Underestimated emergence of persistent changes to TN90p (hot nights) and TX90p (hot days) in CMIP5 ‘Historical’ simulations is linked to a combination of biases in the simulated change (‘signal’) and the simulated variability (‘noise’). Panels show the fraction of CMIP5 ‘Historical’ simulations with signal (**a**,**b**), noise (**c**,**d**) and signal-to-noise ratio (**e**,**f**) of TN90p (left panel) and TX90p (right panel) that would result in a delay of emergence or produce emergence in the opposite direction to observed (see Method). Signal is approximated as the absolute total linear trend in temperature extremes over 1921–2005 and noise as the standard deviation of residuals after removing this linear trend. Stippling indicates where the linear trend in HadEX2 observations is consistent with cooling rather than warming (i.e., a negative trend for TN90p and TX90p). Gray color marks regions for which there is no emergence in HadEX2 observations by the year 2000. White regions have no data. See Fig. S10 for TN10p (cold nights) and TX10p (cold days). The map is produced using R version 3.0.3 software (https://www.r-project.org/).

The observed persistent changes to temperature extremes represent a shift towards more hot days and nights, and fewer cold days and nights, consistent with first-order expectations for a warming world. There are some exceptions to the direction of these shifts (grid cells shaded with cool colors in Fig. 1a,b, and stippled in Fig. 2), such as parts of the ‘warming hole’ in the southeast/central USA^20^, where cold days and nights have increased while hot days and nights have decreased. Several factors could potentially contribute to the ‘warming hole’, such as anthropogenic aerosol emissions, land cover change, unforced internal climate variability (e.g., North Atlantic Oscillation and Pacific Decadal Oscillation) or a combination thereof^22–24^. With the continued emissions of greenhouse gases and the reduction of anthropogenic aerosol emissions, warming in this region is expected to increase in future decades^22^.

Consistent with some earlier studies^25,26^, we find that the changes in the distribution of temperature extremes are primarily the result of a significant shift in the center of the distribution of the examined temperature extremes, rather than a change in the variability of temperature extremes, as reflected by the nearly identical patterns of emergence of persistent changes in the mean of the distribution of temperature extremes (Fig. S5), and the lack of emergence of persistent changes in the variance of the distribution of temperature extremes (Fig. S6). (See captions of Figs S5,S6 for the approaches used for the detection of persistent changes in the mean and variance of the distribution of temperature extremes, respectively.)

We find that climate models largely underestimate how quickly persistent changes in the distribution of temperature extremes have emerged in the direction consistent with warming during the historical period, or fail to represent emergence consistent with cooling (such as the emergence in TX90p over the U.S. ‘warming hole’) (Fig. 1c,d for TN90p and TX90p and Fig S2c,d for TN10p and TX10p). The ensemble median TOE calculated from the CMIP5 ‘Historical’ simulations fails to show emergence by the year 2000 over almost all land area covered by the HadEX2 observations (Fig. S7). In fact, on average across the ‘Historical’ ensemble members, 19–30% of the land with data coverage exhibits emergence of persistent changes consistent with warming (28% for TN90p, 18% for TX90p, 30% for TN10p, and 19% for TX10p). These percentages are considerably smaller than those in the observations, with the exception of TX90p (55% for TN90p, 16% for TX90p, 67% for TN10p, and 28% for TX10p). Furthermore, over 74–92% of the land where persistent changes have already occurred in HadEX2 (74% for TN90p, 92% for TX90p, 83% for TN10p, and 91% for TX10p), more than 80% of the ensemble members in the ‘Historical’ simulations either exhibit a delay of emergence or show emergence in a direction opposite to that observed (Fig. 1c,d and Fig. S2c,d). It is noted that over the regions where persistent changes have already occurred, the simulated emergence that is opposite to the observed direction occurs in a few ensemble members only, and mainly in the U.S. ‘warming hole’ (Fig. S8). Similar underestimation of persistent changes is also found in annual mean temperature (Fig. S4).

We find that the discrepancy between the observed and simulated TOE is unlikely to be caused by internal climate variability (Fig. S9; see the descriptions in the figure caption for the approaches used for statistical tests of this sort). Rather, it primarily results from the joint effects of biases in the externally forced response (signal) and internal climate variability (noise) (Fig. 2 for TN90p and TX90p and Fig. S10 for TN10p and TX10p). When comparing the observed signal to the simulated ‘Historical’ signal (which are approximated by the absolute total linear trends in temperature extremes over the period 1921–2005), we find a marked underestimation over a large majority of land where persistent changes have already occurred (Fig. 2a,b and Fig. S10a,b). Comparing observed noise to simulated noise (which is approximated by the standard deviation of residuals after removing a linear trend) reveals a consistent overestimation throughout the emerged domain, with more than 90% of the ensemble members exhibiting excessive noise over almost all land area covered by the HadEX2 observations (Fig. 2c,d and Fig. S10c,d), which could be due to overly strong land-atmosphere feedbacks in climate models^27^. Taken together, the signal-to-noise ratio is underestimated (Fig. 2e,f and Fig. S10e,f), implying that the simulated temperature extremes require a longer time to exceed the internal variability than is seen in observations. Over the U.S. ‘warming hole’ where the observed emergence has occurred toward cooling, we find fewer than 10% of the ensemble members having a cooling signal that is as strong or stronger than observed. As a result, models are unable to capture the observed emergence in this region as well.

Although the CMIP5 ensemble exhibits biases in both the simulated signal and the simulated noise, biases in the simulated signal appear to play a larger role in delaying the TOE (Figs S11,S12). Based on the ‘Historical’ simulations, only 17–36% of the observed emergence area (36% for TN90p, 17% for TX90p, 28% for TN10p, and 18% for TX10p) is captured by the simulations (i.e., the observed TOE falls in the 16–84% range of the simulated TOE, which is equivalent to ± *σ* for a Gaussian distribution but is more suitable for measuring the dispersion of a non-Gaussian distribution, such as the right-truncated distribution of TOE at 2000, where *σ* is the standard deviation of the Gaussian distribution^19^). This percentage increases to 51–75% (75% for TN90p, 51% for TX90p, 63% for TN10p, and 67% for TX10p) when correcting for the bias in signal (Fig. S9a,d), but only increases to 32–67% (67% for TN90p, 44% for TX90p, 48% for TN10p, and 32% for TX10p) when correcting for the bias in noise (Fig. S12a,d; see Methods). Furthermore, we estimate that over the land where more than 84% of the ensemble members in the ‘Historical’ simulations exhibit a delay of emergence (i.e., fail to show emergence by the year 2000 or exhibit a later TOE than observed), biases in the signal have delayed the emergence by ~1–2 decades (Fig. S11e,f). In contrast, biases in the noise have delayed the emergence by <1 decade (Fig. S12e,f). These results imply that improvement in the simulation of the externally forced response is likely to yield the greatest improvement in prediction of the TOE, although the role of internal variability should not be neglected, especially for regions where the externally forced response is relatively weak. These results also suggest the potential benefits of bias correction procedures that can reduce uncertainties in the projected TOE of future persistent changes to temperature extremes.

**Figure 3:**
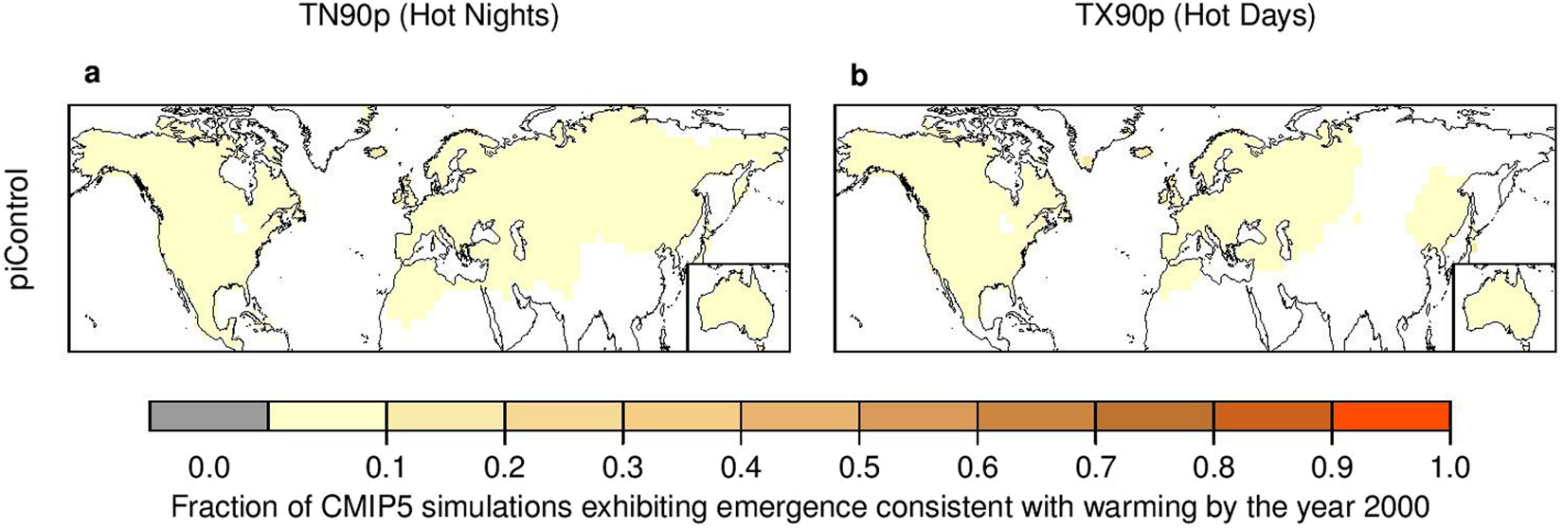
Emergence of persistent changes to TN90p (hot nights) and TX90p (hot days), cannot be explained by natural external forcing, but is likely due to anthropogenic influence, especially anthropogenic emissions greenhouse gases. Panels show the fraction of simulations exhibiting emergence consistent with warming by the year 2000 in ‘HistoricalNat’ simulations (**a**,**b**), ‘Historical’ simulations (**c**,**d**) and ‘HistoricalGHG’ simulations (**e**,**f**) of TN90p (left panels) and TX90p (right panels). White regions have no data. See Fig. S14 for TN10p (cold nights) and TX10p (cold days). The map is produced using R version 3.0.3 software (https://www.r-project.org/).

Although internal climate variability may delay or accelerate the emergence of persistent changes to a climate variable^4,19^, internal variability alone is unlikely to have caused the observed persistent changes in the distribution of daily-scale temperature extremes (Fig. 3 for TN90p and TX90p and Fig. S13 for TN10p and TX10p). To test the role of internal variability in creating persistent changes, we implement our analysis on an ensemble of 540 85-year time series of temperature extremes, drawn from the bias-corrected ‘piControl’ simulations using a block-bootstrap approach (see Methods). The 85-year block-bootstrap is designed to mimic the length of the 1921–2005 historical period, and to maintain the spatial-temporal correlations of temperature extreme fields. Since the overestimation of internal variability (Fig. 2c,d and Fig. S10c,d) may lead to an underestimation of the chance of temporary emergence induced by internal variability, a bias correction procedure is implemented to adjust the simulated internal variability to be consistent in magnitude with the HadEX2 observations. It is found that the likelihood of spurious emergence due to internal variability alone is less than 5% (Fig. 3 and Fig. S13), implying that the observed changes are unlikely to have arisen from internal variability alone.

**Figure 4:**
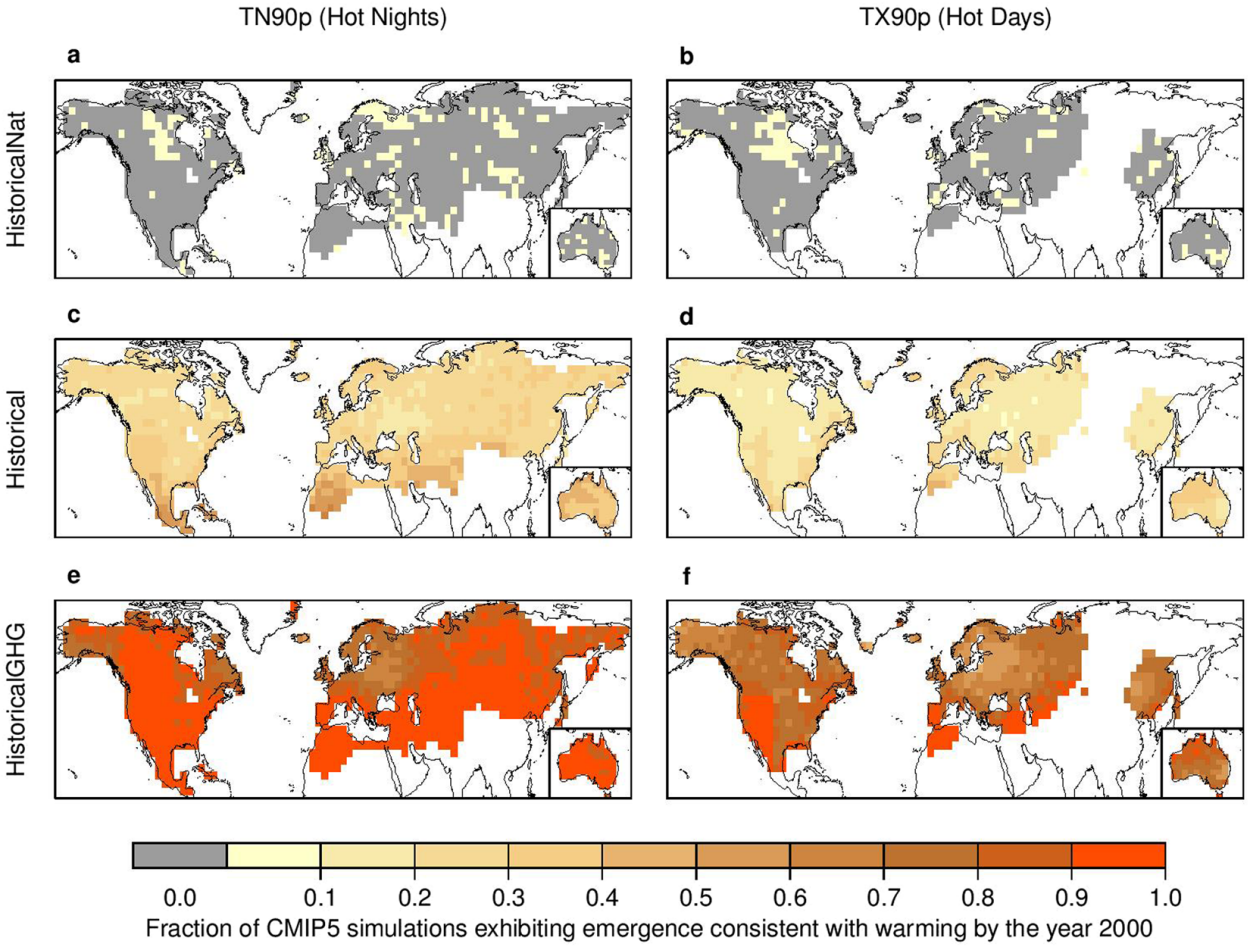
Emergence of persistent changes to TN90p (hot nights) and TX90p (hot days) is unlikely to be explained by internal variability alone. Panels show the fraction of simulations exhibiting emergence consistent with warming by the year 2000 in an ensemble of 540 85-year time series of TN90p (**a**) and TX90p (**b**) drawn from the bias-corrected ‘piControl’ simulations in terms of a block-bootstrap approach to mimic the length of the 1921–2005 historical period (see Methods). A bias correction is implemented to adjust the simulated internal variability to be consistent in magnitude with the HadEX2 observations. White regions have no data. See Fig. S13 for TN10p (cold nights) and TX10p (cold days). The map is produced using R version 3.0.3 software (https://www.r-project.org/).

Similarly, persistent emergence is not detected by the end of the historical period in the ‘HistoricalNat’ simulations, which lack anthropogenic forcings (Fig. 4a,b for TN90p and TX90p and Fig. S14a,b for TN10p and TX10p). In contrast, the ‘HistoricalGHG’ simulations, which are forced only by anthropogenic increases in greenhouse gas concentrations, result in >50% of ensemble members showing emergence consistent with warming over most of the areas of observed emergence, including >90% of such ensemble members over large areas of observed emergence in nighttime extremes (Fig. 4e,f and Fig. S14e,f). Combined with our results that internal variability alone is unlikely to have caused the observed emergence (Fig. 3 and S11), these qualitative comparisons indicate that the historical emergence of persistent changes in temperature extremes consistent with warming is more likely to be anthropogenic than natural in origin. Our results are in agreement with the optimal fingerprinting-based attribution studies of absolute changes in the examined extreme temperature indices (e.g., trend)^28,29^, as well as extreme event attribution studies based on a similar emergence analysis^30^, thus adding to an increasing body of evidence of the anthropogenic influence on temperature extremes. Based on the present analysis, however, we could not ascertain whether the detected emergence in the U.S. ‘warming hole’ is due to anthropogenic activity.

Our model assessment focuses mainly on regions where persistent changes had already occurred by the year 2000. In regions where persistent changes had not emerged up to the year 2000, we find that over half of the ensemble members of the ‘Historical’ simulations also do not show emergence (Fig. S7). On the other hand, we find that the ratios of the observed 1921–2005 linear trends to the noise of climate variability are generally consistent with the 16–84% range of the simulations over most parts of these regions (Fig. S15). We therefore expect that future emergence of persistent changes to temperature extremes in these regions is likely to be reasonably simulated by climate models. Moreover, due to the lack of sufficient observational data in the tropics and over large portions of the southern hemisphere, at present we cannot access observed TOE and model performance in these regions. In this sense, our analysis is restricted by the observational data availability to the northern hemispheric land areas with relatively lower signal-to-noise ratio for temperature extremes.

We note that the reported underestimation of signal in temperature extremes over many parts of the land where persistent changes have already occurred is not inconsistent with existing studies^20,28,29,31–33^, which report that in general climate models reproduce the observed temperature extremes reasonably well at the global scale, but may underestimate changes in different extreme temperature indices in some sub-continental regions.

## Conclusions

Overall, our analysis shows that widespread persistent changes in the distribution of daily-scale temperature extremes have already occurred over large parts of the Earth, and that these observed changes are likely due to anthropogenic influence, especially the historical increase of anthropogenic emissions of greenhouse gases. Further, the fact that the emergence of these changes is delayed over many areas in the CMIP5 ‘Historical’ simulations suggests that those models may also underestimate the speed and spatial extent of future changes in the distribution of temperature extremes over parts of the Earth.

## Methods

We focus on four extreme temperature indices defined by the Expert Team on Climate Change Detection and Indices^15^ (i.e., TN90p, TX90p, TN10p and TX10p) from the HadEX2 3.75° × 2.5° gridded observations for 1921–2005^13^, along with a suite of CMIP5 climate model simulations over the same period and the corresponding long pre-industrial control simulations. Extreme indices calculated on the model’s native grids^34^ are interpolated to the grid of HadEX2 using bilinear interpolation. For each extreme index, we restrict our analysis to grid cells with at most 5 years of missing observations.

To assess whether persistent changes in the distribution of temperature extremes have already occurred, we estimate TOE from the HadEX2 observations for each extreme index at each grid cell. If the TOE occurs in 2000 or earlier, we further note whether or not the emergence is consistent with warming or cooling; otherwise, we consider that no persistent change occurred (Fig. 1a–b). To assess whether the observed emergence is captured by climate models, for each extreme index at each grid cell where emergence has already occurred in the HadEX2 observations, we calculate the fraction CMIP5 ‘Historical’ simulations that either fail to show emergence by the year 2000, or exhibit a delay in the TOE, or show emergence in the direction opposite to observed (Fig. 1c–d). We note that simulated emergence in a direction opposite to observed is found mainly in a few ensemble members in U.S. the ‘warming hole’ (Fig. S8).

We assess errors in the simulated emergence by analyzing biases in the simulated change (‘signal’) and the simulated variability (‘noise’). For each extreme index at each grid cell where emergence has already occurred in HadEX2, we calculate the fractions of the ‘Historical’ simulations with signal, noise, and signal-to-noise ratio that would result in a delay of emergence or produce emergence in the direction opposite to observed (Fig. 2). We approximate the signal as the absolute total linear trend in a given extreme index over 1921–2005, and the noise as the standard deviation of residuals after removing this linear trend.

To separate the role of biases in the simulated signal and the simulated noise in delaying the TOE, we assume that extreme temperature indices can be decomposed into externally forced response (‘signal’) and internal climate variability (‘noise’). A robust LOESS-fit with a 40-year moving window is used to separate signal and noise, which accounts for the nonlinear evolution of the extreme temperature indices^35,36^. For each member in the ‘Historical’ simulations, we scale the simulated signal such that it has the same linear trend as observed and replace the native signal with the observed signal, and we scale the simulated noise such that it has the same standard deviation as observed. Consequently, we obtain an ensemble of bias-corrected ‘Historical’ simulations, in which biases in either the signal or the noise are corrected. We implement the TOE estimation on the bias-corrected simulations. For each extreme index at each grid cell where emergence has already occurred in the HadEX2 observations, we calculate the ensemble median difference in the TOE before and after bias correction, to estimate the number of years by which biases in the signal or noise have delayed the TOE. In order to obtain robust estimates, we exclude simulations that do not show emergence by the year 2000 in either the native or the bias-corrected simulations, and exclude those with emergence occurring in opposite directions before and after bias correction. We also calculate the fraction of the observed emergence area with the observed TOE falling in the 16–84% ranges of the TOE derived from constructed simulations, to quantify how much of the observed emergence would be captured if there were no bias in the signal or noise.

To assess the possibility that the observed emergence could occur due to internal climate variability alone, we analyze ‘piControl’ simulations from 27 climate models. All ‘piControl’ extreme indices are linearly detrended to limit the impact of model drift, and then scaled such that the standard deviations of the detrended indices are of the same magnitude as observed. For each extreme index, we draw 540 samples of 85-year time series (i.e., 20 samples from each of the 27 climate models) using a block-bootstrap approach, which is implemented as follows: We first draw a sample of 85 consecutive years with replacement from the long simulations and then simultaneously draw the simulations at all grid cells falling in this time period. In doing so, the spatial-temporal autocorrelations in the extreme indices are preserved. The 85-year period is designed to mimic the length of the 1921–2005 historical period. We then implement our detection procedure on these 540 samples and calculate the fraction of samples showing emergence consistent with warming by the year 2000 (i.e., 5 years before the end of the record; Fig. 3).

We also explore whether or not the observed emergence is more likely to be anthropogenic in origin rather than due to natural external forcings, by comparing the fraction of the ‘HistoricalNat’, ‘Historical’ and ‘HistricalGHG’ simulations exhibiting emergence consistent with warming by the year 2000 over the 1921–2005 historical period (Fig. 4).

## Electronic supplementary material

Supporting materials

## References

[1] Field, C. B. *et al*. Ed. Managing the Risk of Extreme Events and Disasters to Advance Climate Change Adaptation, Cambridge Univ. Press, New York, USA.

[2] Diffenbaugh NS, Scherer M (2011). Observational and model evidence of global emergence permanent, unprecedented heat in the 20^th^ and 21st centuries. Climatic Change.

[3] Scherer M, Diffenbaugh NS (2014). Transient twenty-first century changes in daily-scale temperature extremes in the United States. Clim. Dyn..

[4] King AD (2015). The timing of anthropogenic emergence in simulated climate extremes. Environ. Res. Lett..

[5] Bador, M., Terray, L. & Boé J. Emergence of human influence on summer record breaking temperatures over Europe. *Geophys. Res. Lett*. **43** (2015).

[6] Mahlstein I, Knutti R, Solomon S, Portmann RW (2011). Early onset of significant local warming in low latitude countries. Environ. Res. Lett..

[7] Hawkins E, Sutton R (2012). Time of emergence of climate signals. Geophys. Res. Lett..

[8] Giorgi F, Bi X (2009). Time of emergence (TOE) of GHG-forced precipitation change hot-spots. Geophys. Res. Lett..

[9] Mahlstein I (2012). Perceptible changes in regional precipitation in a future climate. Geophys. Res. Lett..

[10] Lyu K (2014). Time of emergence for regional sea-level change. Nature Clim. Change.

[11] Fyke JG, Vizaíno M, Lipscomb WH (2014). The pattern of anthropogenic signal emergence in Greenland Ice Sheet surface mass balance. Geophys. Res. Lett..

[12] Mahlstein I, Hegerl G, Solomon S (2012). Emerging local warming signals in observational data. Geophys. Res. Lett..

[13] Donat MG (2013). Updated analyses of temperature and precipitation extreme indices since the beginning of the twentieth century: The HadEX2 dataset. J. Geophys. Res. Atmos..

[14] Taylor KE, Stouffer RK, Meehl GA (2012). An overview of CMIP5 and the experiment design. Bull. Am. Meteorol. Soc..

[15] Zhang X (2011). Indices for monitoring changes in extremes based on daily temperature and precipitation data. WIREs Clim. Change.

[16] Simolo C, Brunetti M, Maugeri M, Nanni T (2011). Evolution of extreme temperatures in a warming climate. Geophys. Res. Lett..

[17] Åström DO, Forsberg B, Ebi KL, Rocklöv J (2013). Attributing mortality from extreme temperatures to climate change in Stockholm, Sweden. Nature Clim. Change.

[18] Klein, T. A., Zwiers, F. W. & Zhang X. Guidelines on analysis of extremes in a changing climate on support of informed decisions for adaptation. *Report, World Climate Data and Monitoring Programme (WCDMP) series, WCDMP-72*.

[19] Hawkins E (2014). Uncertainties in the timing of unprecedented climates. Nature.

[20] Bindoff, N. L. *et al*. in *Climate Change2013: The physicalScience Basis (*eds Stocker, T. E. *et al*.) Ch. 10 (IPCC, Cambridge Univ. Press, 2013).

[21] Simolo C, Brunetti M, Maugeri M, Nanni T, Speranza A (2010). Understanding climate change-induced variations in daily temperature distribution over Italy. J. Geophys. Res. Atmos..

[22] Mascioli NR, Previdi M, Fiore AM, Ting M (2017). Timing and seasonality of the United States ‘warming hole’. Environ. Res. Lett..

[23] Yu S (2014). Attribution of the United States ‘warming hole’: Aerosol indirect effect and precipitable water vapor. Sci. Rep..

[24] Diffenbaugh NS (2009). Influence of modern land cover on the climate of the United States. Clim. Dyn..

[25] Donat MG, Alexander LV (2012). The shifting probability distribution of global daytime and night-time temperatures. Geophys. Res. Lett..

[26] Weaver SJ, Kumar A, Chen M (2014). Recent increases in extreme temperature occurrence over land. Geophys. Res. Lett..

[27] Dirmeyer PA, Koster RD, Guo Z (2006). Do global models properly represent the feedback between land and atmosphere?. J. Hydrometeor..

[28] Morak S, Hegerl GC, Kenyon J (2011). Detectable regional changes in the number of warm nights. Geophys. Res. Lett..

[29] Morak S, Hegerl GC, Christidis D (2013). Detectable changes in the frequency of temperature extremes. J. Clim..

[30] Diffenbaugh N (2016). Quantifying the influence of global warming on unprecedented extreme climate events. Proc. Natl. Acad. Sci. USA.

[31] Zwiers F, Zhang X, Feng Y (2011). Anthropogenic influence of long return period daily temperature extremes at regional scales. J. Clim..

[32] Min SK (2013). Multimodel detection and attribution of extreme temperature changes. J. Clim..

[33] Wang Z, Jiang Y, Wan H, Yan J, Zhang X (2017). Detection and attribution of changes in extreme temperatures at regional scale. J. Clim..

[34] Sillmann J (2013). Climate extremes indices in the CMIP5 multimodel ensemble: Part 1. Model evaluation in the present climate. J. Geophys. Res. Atmos..

[35] Loader, C. *Local Regression and Likelihood*. Springer, New York (1999).

[36] Mahlstein I, Spirig C, Liniger MA, Appenzeller C (2015). Estimating daily climatologies for climate indices derived from climate model data and observations. J. Geophys. Res. Atmos..

