# Supplementary material for: Widespread persistent changes to temperature extremes occurred earlier than predicted
Source: Sci Rep. 2018 Jan 17;8:1007. doi: 10.1038/s41598-018-19288-z (PMC5772442; doi:10.1038/s41598-018-19288-z)
Supplement: Supplementary file 1 — Supporting materials [file 41598_2018_19288_MOESM1_ESM.pdf]

Widespread persistent changes to temperature extremes occurred earlier than predicted

Chao Li[1,2*], Yuanyuan Fang[1], Ken Calderia[1], Xuebin Zhang[3], Noah S. Diffenbaugh[4,5], and Anna M. Michalak[1]

[1]Department of Global Ecology, Carnegie Institution for Science, Stanford, California, 94305, USA

[2]Pacific Climate Impacts Consortium, University of Victoria, Victoria, British Columbia, V8W 2Y2, Canada

[3]Climate Research Division, Environment and Climate Change Canada, Toronto, Ontario, M3H 5T4, Canada

[4]Deparment of Earth System Science, Stanford University, Stanford, California, 94305, USA

[5]Woods Institute for the Environment, Stanford University, Stanford, California, 94305, USA

*Corresponding to: chaoli@uvic.ca

Supporting Online File

List of contents:

Table S1

Table S2

Figure S1

Figure S2

Figure S3

Figure S4

Figure S5

Figure S6

Figure S7

Figure S8

Figure S9

Figure S10

Figure S11

Figure S12

Figure S13

Figure S14

Figure S15

Table S1: Simulations and ensemble sizes from CMIP5 climate models analyzed in this study. The symbol '✓' indicates that 'piControl' simulations from the corresponding models are analyzed.

CMIP5 models	'Historical'	'HistoricalGHG'	'HistoricalNat'	'piControl'
ACCESS1-0	1			
bcc-csm1-1	3	1	1	✓
bcc-csm1-1-m	3			✓
CanESM2	5	5	5	✓
CCSM4	2	3	2	✓
CESM1-FASTCHEM	3			
CESM1-CAM5-1-FV2		2		
CMCC-CESM	1			✓
CMCC-CM	1			✓
CMCC-CMS	1			✓
CNRM-CM5	10	6	6	✓
CSIRO-Mk3-6-0	10	5	5	✓
EC-EARTH	2			
FGOALS-s2	3			✓
GFDL-CM3	5	3	3	✓
GFDL-ESM2G	1			✓
GFDL-ESM2M	1	1	1	✓
GISS-E2-R	3			
HadCM3	10			✓
HadGEM2-CC	1			✓
HadGEM2-ES	4	4	4	✓
inmcm4	1			
IPSL-CM5A-LR	6	6	3	✓
IPSL-CM5A-MR	3	3	3	✓
IPSL-CM5B-LR	1			✓
MIROC5	5			✓
MIROC-ESM	3	3		✓
MIROC-ESM-CHEM	1	1	1	✓
MPI-ESM-LR	3			✓
MPI-ESM-MR	3			✓
MPI-ESM-P	2			✓
MRI-CGCM3	5	1	1	✓
NorESM1-M	2	1	1	✓

Table S2: Four indices representative of temperature extremes recommended by the Expert Team on Climate Change and Indices. Precise definitions can be found at http://etccdi.pacificclimate.org/list_27_indices.shtml.

ID	Index name	Index definition	Units
TN10p	Cold nights	Annual percentage of days when daily minimum temperature < 10th percentile of the baseline 1961-90 period	%
TN90p	Hot nights	Annual percentage of days when daily minimum temperature > 90th percentile of the baseline 1961-90 period	%
TX10p	Cold days	Annual percentage of days when daily maximum temperature < 10th percentile of the baseline 1961-90 period	%
TX90p	Hot days	Annual percentage of days when daily maximum temperature > 90th percentile of the baseline 1961-90 period	%

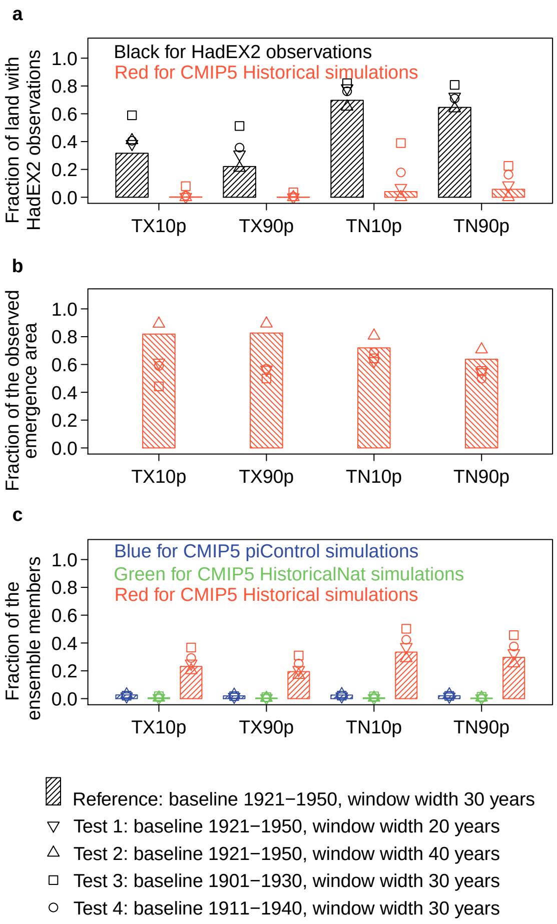

Figure S1: Major findings reported in this study are robust to the selection of different baseline periods and moving window widths. Although the time of emergence (TOE) of persistent changes to temperature extremes depends to some extent on the baseline period and the moving window width, the spatial patterns of emergence are insensitive to the baseline period or the moving window width, consistent with existing studies on persistent changes to summer mean temperatures[4]. Moreover, the selection of different baseline periods or moving window widths does not change the major findings of this study. **a**) The fraction of the land covered by the HadEX2 observations showing emergence of persistent changes to TX10p, TX90p, TN10p and TN90p by the year 2000 based on the TOE derived from the HadEX2 observations (black) and the ensemble median TOE derived from CMIP5 ‘Historical’ simulations (red). **b**) The fraction of the observed emergence area with more than 84% of the ensemble members in the ‘Historical’ simulations showing no emergence by the year 2000 or exhibiting a delay in the TOE. **c**) The area-weighted average of the fraction of ensemble members showing emergence consistent with warming by the year 2000 in an ensemble of 540 85-year time series of temperature extremes drawn from bias-corrected ‘piControl’ simulations (blue; see Methods), in ‘HistoricalNat’ simulations (green) and ‘Historical’ simulations (red) over the land with the HadEX2 observations. Bars, inverted triangles, triangles, squares and circles are for results derived with the reference baseline period of 1921-1950 and moving window width of 30 years, a baseline period of 1921-1950 and a moving window width of 20 years, a baseline period of 1921-1950 and a moving window width of 40 years, a baseline period of 1901-1930 and a moving window width of 30 years, and a baseline period of 1911-1940 and a moving window width of 30 years, respectively.

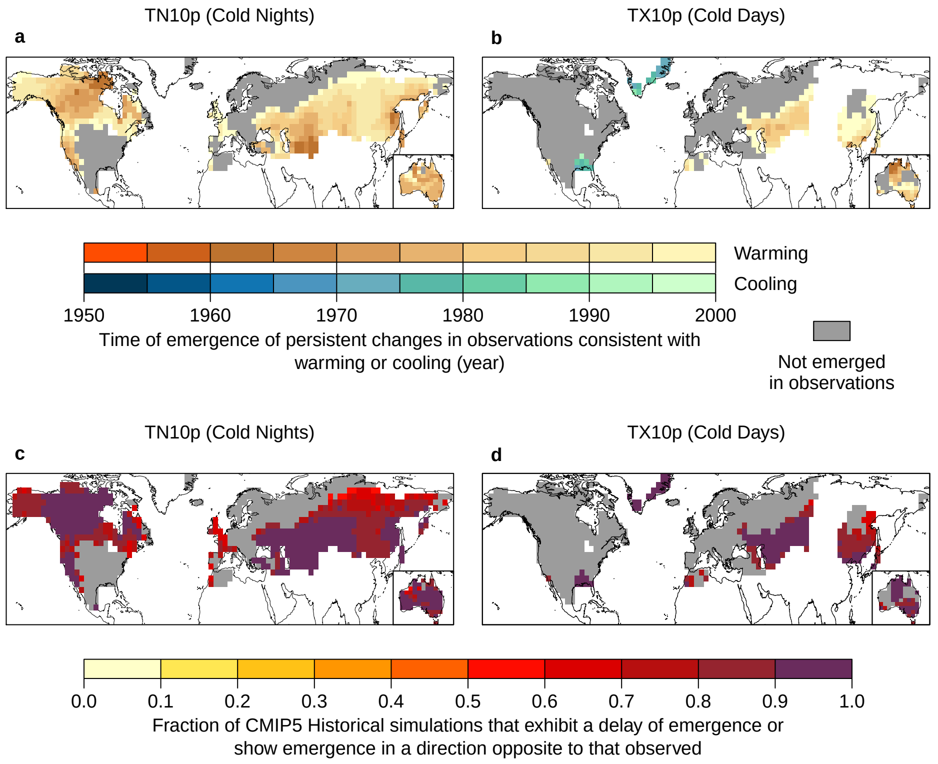

Figure S2: Persistent changes to TN10p (cold nights) and TX10p (cold days) have already occurred over large parts of the Earth and climate models underestimate these persistent changes. Top panels show time of emergence (TOE) of persistent changes to TN10p (**a**) and TX10p (**b**) derived from HadEX2 observations. Warm (cool) color marks regions where the emergence of persistent changes occurs in the direction consistent with warming (cooling). Gray color marks regions for which there is no emergence in HadEX2 observations by the year 2000. White regions have no data. See Fig S7 for CMIP5 results corresponding to these panels. Bottom panels show the fraction of CMIP5 'Historical' simulations that exhibit a delay of emergence of persistent changes to TN10p (**c**) and TX10p (**d**) or show emergence in a direction opposite to that observed (see Methods). It is noted that simulated emergence in the opposite direction to observed is restricted mainly to the 'warming hole' in southeast/central USA and to a few ensemble members (see Fig S8). The map is produced using R version 3.0.3 software (https://www.r-project.org/).

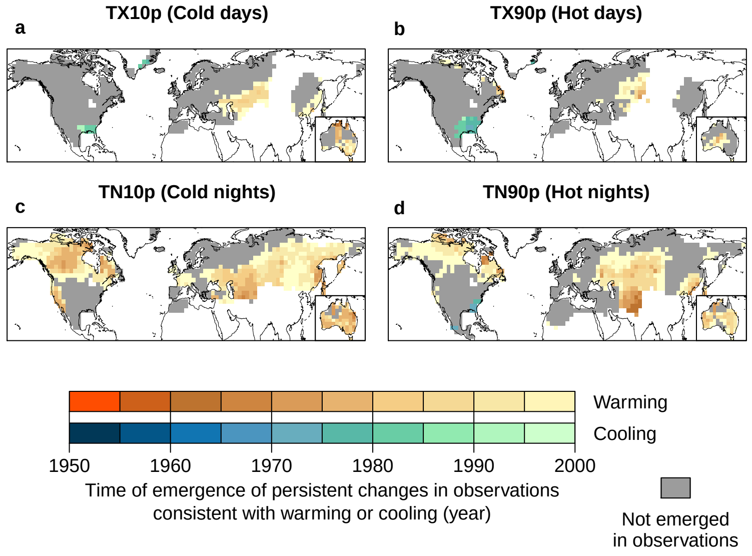

Figure S3: Autocorrelation in temperature extremes plays a limited role in the detected emergence of persistent changes to TX10p (cold days), TX90p (hot days), TN10p (cold nights) and TN90p (hot nights). Panels show time of emergence of persistent changes to TX10p (**a**), TX90p (**b**), TN10p (**c**) and TN90p (**d**) derived from HadEX2 observations, using a 5-year block-bootstrap K-S test rather than the traditional K-S test in the detection procedure. Warm (cool) color marks regions where the emergence of persistent changes occurs in the direction consistent with warming (cooling). Gray color marks regions for which there is no emergence in HadEX2 observations by the year 2000. White regions have no data. Given two time series of an extreme index from two 30-year periods, denoted respectively by X_1 and X_2, the 5-year block-bootstrap K-S test, with a null hypothesis that X_1 and X_2 come from the same distribution proceeds as follows. Step 1: calculate the K-S statistic using X_1 and X_2 and denote by K. Step 2: draw two samples, each with a size of 30, from the concatenated series of X_1 and X_2, using 5-year block-sampling with replacement, and calculate the K-S statistic using the two samples thus obtained. Step 3: repeat Step 2 for 1000 times and denote the obtained differences as K_1, K_2, $\ldots$, K_{1000}. Step 4: determine whether K is greater than the 0.95-quantile of K_1, K_2, $\ldots$, K_{1000}; if so, the null hypothesis can be rejected at significance level 5% and thus X_1 and X_2 come from different distributions; otherwise, the null hypothesis cannot be rejected. The map is produced using R version 3.0.3 software (https://www.r-project.org/).

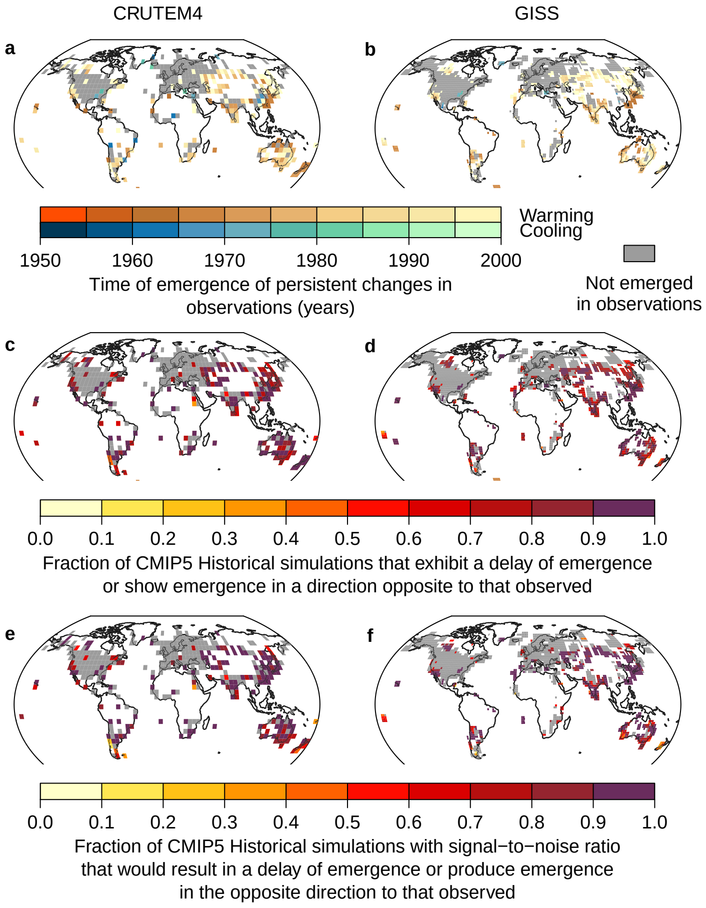

Figure S4: Persistent changes to temperature extremes, especially the night temperature extremes, tend to emerge more widespread and earlier than persistent changes to annual mean temperature. Top panels show time of emergence (TOE) of persistent changes to annual mean temperature from CRUTEM4 (**a**) and GISS observations (**b**), respectively. Warm (cool) color marks regions where the emergence of persistent changes occurs in the direction consistent with warming (cooling). Middle panels show the fraction of CMIP5 'Historical' simulations that exhibit a delay of emergence of persistent changes to annual mean temperature compared respectively to CRUTEM4 (**c**) and GISS observations (**d**) or show emergence in a direction opposite to that observed. Bottom panels show the fraction of CMIP5 'Historical' simulations with signal-to-noise ratio of annual mean temperature that would result in a delay of emergence compared respectively to CRUTEM4 (**e**) and GISS observations (**f**) or produce emergence in the opposite direction to observed. Gray color marks regions for which there is no emergence in the observations by the year 2000. White regions have no data. The map is produced using R version 3.0.3 software (https://www.r-project.org/).

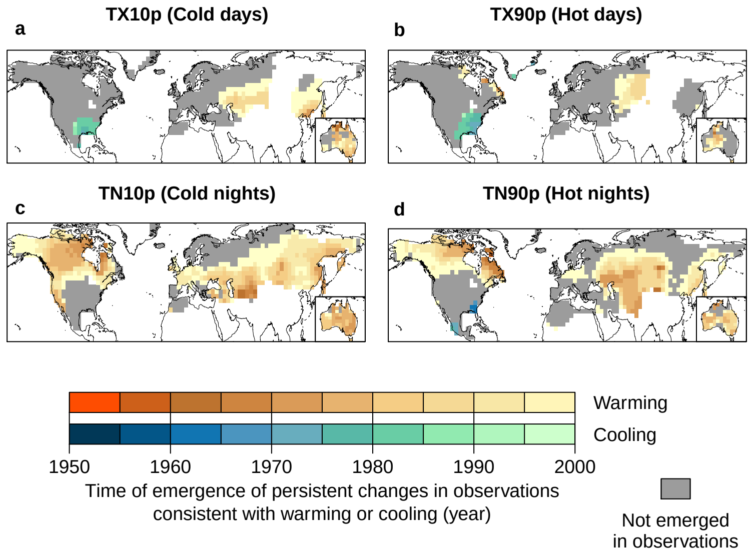

Figure S5: Emergence of persistent changes in the mean of the distributions of TX10p (cold days), TX90p (hot days), TN10p (cold nights) and TN90p (hot nights) is nearly identical to the patterns of emergence of persistent changes in the overall distribution. Panels show the time of emergence of persistent changes in the mean of the distributions of TX90p (**a**), TN10p (**b**), TX10p (**c**) and TN90p (**d**) derived from HadEX2 observations, using a 5-year block-bootstrap test for change in the mean with 5% significance. Warm (cool) color marks regions where the emergence of persistent changes occurs in the direction consistent with warming (cooling). Gray color marks regions for which there is no emergence in HadEX2 observations by the year 2000. White regions have no data. The 5-year block-bootstrap test for change in the mean is implemented following the same procedures as the block-bootstrap K-S test in Fig S3, but replacing the K-S statistic in Step 2 with the absolute difference in the sample mean. The map is produced using R version 3.0.3 software (https://www.r-project.org/).

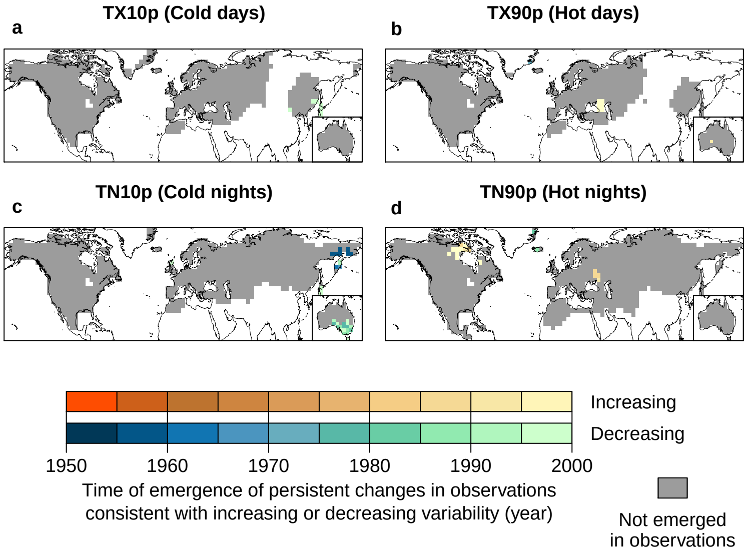

Figure S6: The overall lack of emergence of persistent changes in the variance of the distributions of TX10p (cold days), TX90p (hot days), TN10p (cold nights) and TN90p (hot nights). Panels show the time of emergence of persistent changes in the variance of the distributions of TX90p (**a**), TN10p (**b**), TX10p (**c**) and TN90p (**d**) derived from HadEX2 observations, using a 5-year block-bootstrap test for change in the variance with 5% significance. Warm (cool) color marks regions where the emergence of persistent changes occurs in the direction of positive (negative) trend in the variance. Gray color marks regions for which there is no emergence in HadEX2 observations by the year 2000. The 5-year block-bootstrap test for change in the mean is implemented following the same procedures as the block-bootstrap K-S test in Fig S3, but replacing the K-S statistic in Step 2 with the absolute difference in the sample variance. The map is produced using R version 3.0.3 software (https://www.r-project.org/).

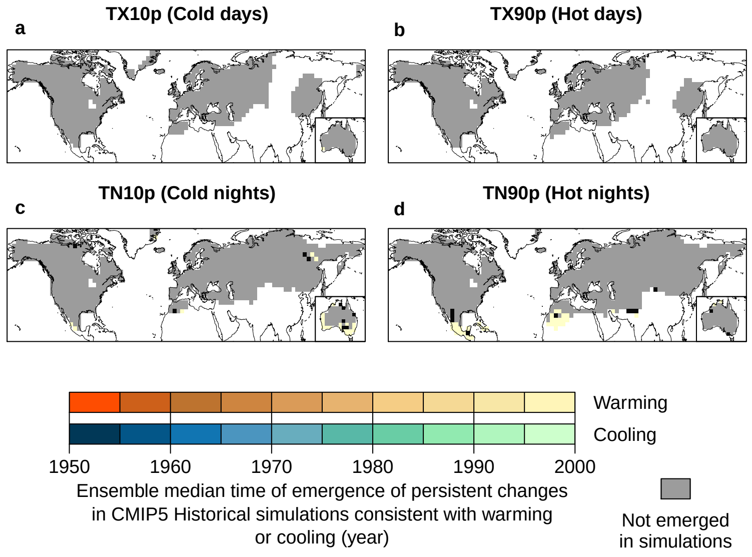

Figure S7: Almost all land coincident with the HadEX2 observations does not show emergence of persistent changes to TX10p (cold days), TX90p (hot days), TN10p (cold nights) and TN90p (hot nights) by the year 2000 based on the ensemble median time of emergence (TOE) calculated from the CMIP5 'Historical' simulations. Panels show ensemble median TOE of persistent changes to TX10p (**a**), TX90p (**b**), TN10p (**c**) and TN90p (**d**) derived from CMIP5 'Historical' simulations. Warm (cool) color marks regions where the ensemble median emergence of persistent changes occurs in the direction consistent with warming (cooling). Gray color marks regions for which there is no emergence by the year 2000 according to the ensemble median TOE. Black color indicates that there is no agreement among simulations. White regions have no data. Given an ensemble of simulations from a suite of climate models, we first estimate TOE for each simulation of each index at each grid cell. If the TOE occurs in 2000 or early, we further note whether or not the emergence is consistent with warming or cooling; otherwise, we consider that no persistent change occurred. By doing so, all simulations can be categorized into three groups: I) simulations that do not show emergence, II) simulations showing emergence consistent with warming, and III) simulations showing emergence consistent with cooling. If none of the three groups contain more than half of the total simulations, then there is no agreement among simulations (e.g., dark gray cells) and thus there is no meaning to calculate the ensemble median TOE; otherwise, the ensemble median TOE is calculated as follows. First, from groups II) and III), we define the one with more simulations as the majority group and the other one as the minority group. We arbitrarily assign a year between 2001 and 2005 as the TOE for each simulation in the minority group. Then the ensemble median TOE is calculated by pooling TOE's from the three groups. Correspondingly, the direction (i.e., warming or cooling) of the ensemble median emergence can be determined. The map is produced using R version 3.0.3 software (https://www.r-project.org/).

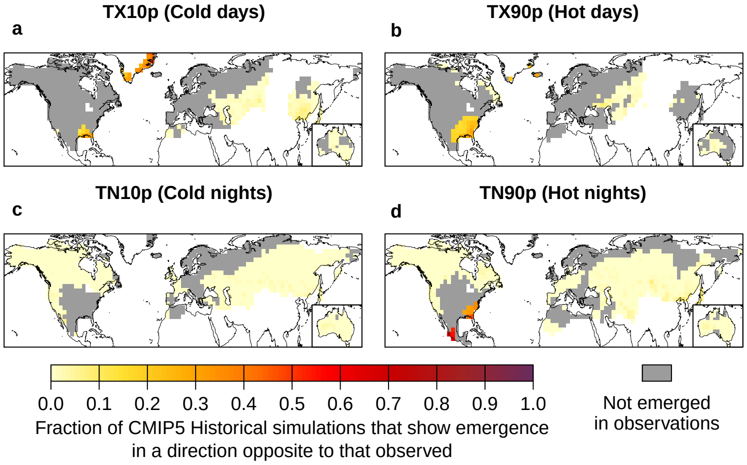

Figure S8: Simulated emergence of persistent changes to to TX10p (cold days), TX90p (hot days), TN10p (cold nights) and TN90p (hot nights) in the opposite direction to observed is found mainly in the U.S. warming hole and in a few ensemble members. Panels show the fraction of CMIP5 Historical simulations exhibiting emergence of persistent changes to TX10p (**a**), TX90p (**b**), TN10p (**c**), and TN90p (**d**) in the opposite direction to observed. Gray color marks regions for which there is no emergence in HadEX2 observations by the year 2000. White regions have no data. The map is produced using R version 3.0.3 software (https://www.r-project.org/).

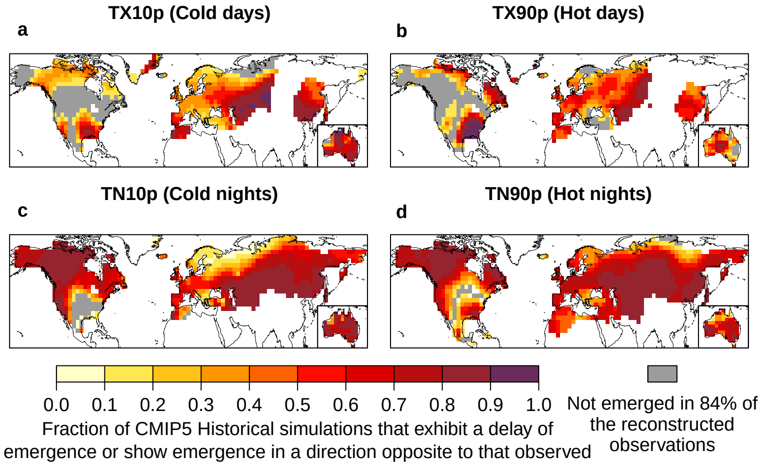

Figure S9: The discrepancy between the observed and simulated time of emergence of persistent changes to TX10p (cold days), TX90p (hot days), TN10p (cold nights) and TN90p (hot nights) is unlikely to be caused by internal climate variability. To explore the role of internal variability in the discrepancy between the observed and simulated TOE, we construct an ensemble of 540 85-year synthetic observations of temperature extremes by superimposing the 540 85-year time series of temperature extremes drawn from bias-corrected 'piControl' simulations on the corresponding externally forced response estimated from HadEX2 observations (see Methods). In doing so, the constructed observations sample more comprehensively the effect of internal variability in the observed temperature extremes. For each extreme index at each grid cell, we implement the emergence detection procedure on each of the constructed observations, calculate the fraction of CMIP5 'Historical' simulations that exhibit a delay of emergence or show emergence in a direction opposite to that in each of the constructed observations, and average the fraction across the ensemble of 540 constructed observations. Panels show the averaged fractions for TX10p (**a**), TX90p (**b**), TN10p (**c**) and TN90p (**d**). Gray color marks regions for which more than 84% of the constructed observations do not show emergence by the year 2000. Obviously, the underestimated persistent changes to temperature extremes by climate models forced with natural and anthropogenic historical forcings cannot be explained by internal climate variability, as reflected by the high averaged fractions (>80%) over a large majority of land where persistent changes have already occurred in the HadEX2 observations. The map is produced using R version 3.0.3 software (https://www.r-project.org/).

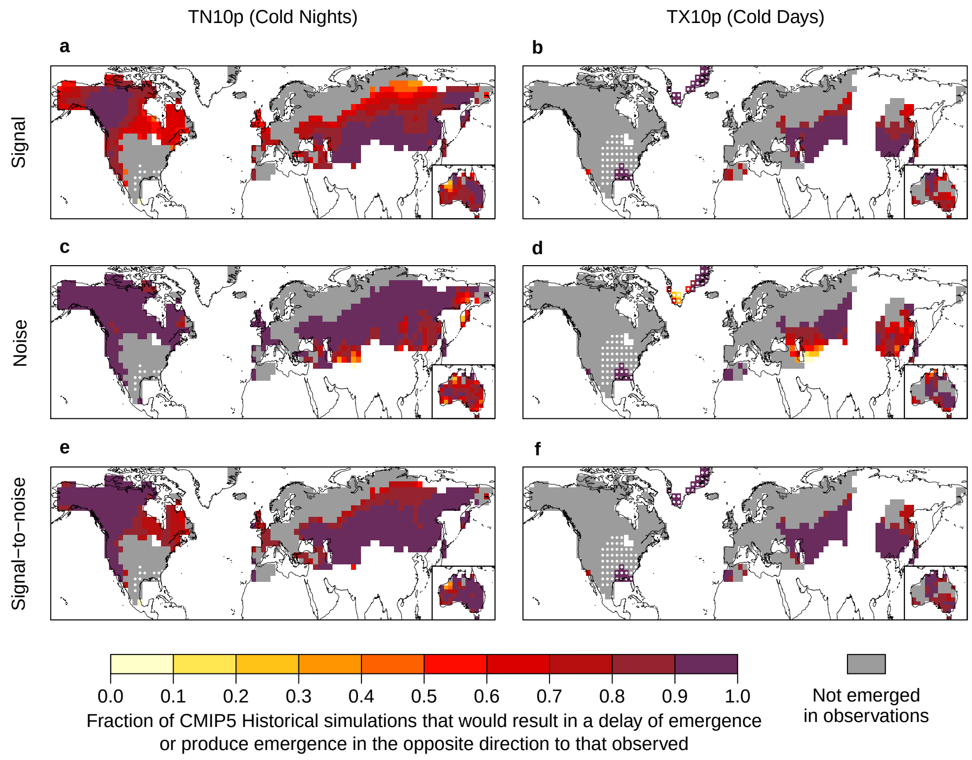

Figure S10: Underestimated emergence of persistent changes to TN10p (cold nights) and TX10p (cold days) in CMIP5 'Historical' simulations is linked to a combination of biases in the simulated change ('signal') and the simulated variability ('noise'). Panels show the fraction of CMIP5 'Historical' simulations with signal (**a-b**), noise (**c-d**) and signal-to-noise ratio (**e-f**) of TN90p (left panel) and TX90p (right panel) that would result in a delay of emergence or produce emergence in the opposite direction to observed (see Method). Signal is approximated as the absolute total linear trend in temperature extremes over 1921-2005 and noise as the standard deviation of residuals after removing this linear trend. Stippling indicates where the linear trend in HadEX2 observations is consistent with cooling rather than warming (i.e., a negative trend for TN90p and TX90p). Gray color marks regions for which there is no emergence in HadEX2 observations by the year 2000. White regions have no data. The map is produced using R version 3.0.3 software (https://www.r-project.org/).

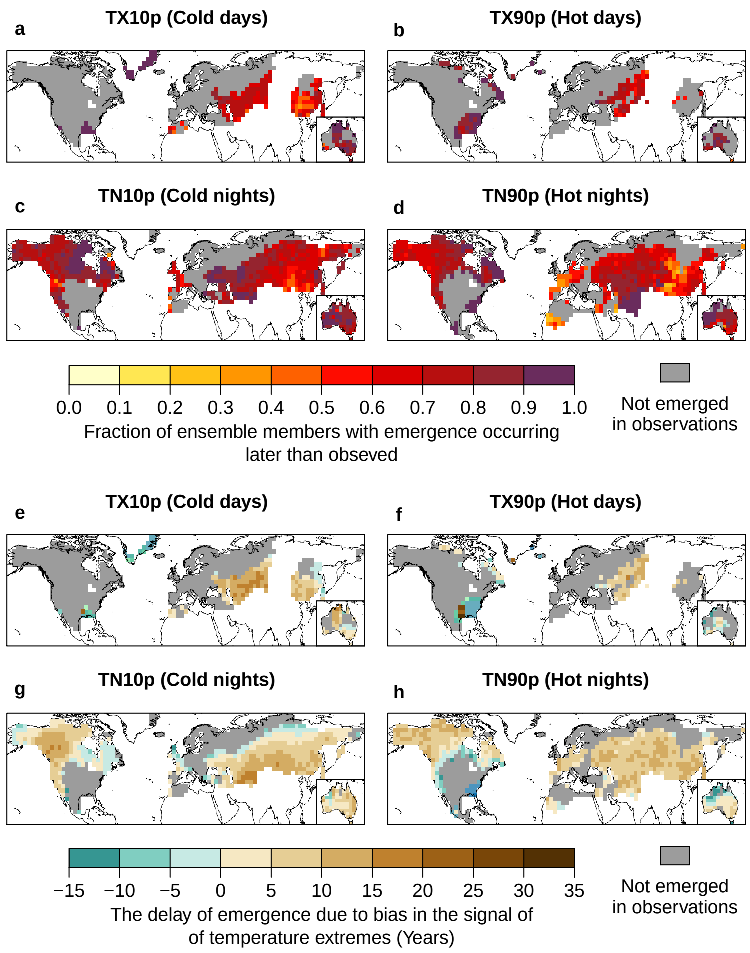

Figure S11: The role of biases in 'signal' in delaying the emergence of persistent changes to TX10p (cold days), TX90p (hot days), TN10p (cold nights) and TN90p (hot nights). Top panels show the fraction of ensemble members in the bias-corrected CMIP5 'Historical' simulations with emergence of persistent changes to TX10p (**a**), TX90p (**b**), TN10p (**c**) and TN90p (**d**) occurring in the same direction as observed but exhibiting a delay in the time of emergence. Comparing to the native CMIP5 'Historical' simulations, the bias in signal is corrected (see Methods). After correcting for the bias in signal, about 50-70% (67% for TX10p, 50% for TX90p, 70% for TN10p and 50% for TN90p) of the observed emergence area is reproduced by the simulations (i.e., the observed TOE falls in the 16-84% range of the simulated TOE, which is equivalent to $\pm\sigma$ for a Gaussian distribution but is more suitable for measuring the dispersion of a non-Gaussian distribution[19]). Bottom panels show the ensemble median difference in TOE of TX10p (**e**), TX90p (**f**), TN10p (**g**) and TN90p (**h**) derived from the native and the bias-corrected CMIP5 'Historical' simulations. To obtain a robust estimate of the ensemble median difference, we exclude the ensemble members with the emergence of persistent changes occurring in different directions before and after bias correction. To reduce the influence of the end-of-data effect, we also exclude the ensemble members that do not show emergence by the year 2000 in either the native or the bias-corrected simulations, and only show results for the regions where persistent changes to temperature extreme have already occurred by the year 2000 in the HadEX2 observations. Gray color marks regions for which there is no emergence in the HadEX2 observations by the year 2000. The map is produced using R version 3.0.3 software (https://www.r-project.org/).

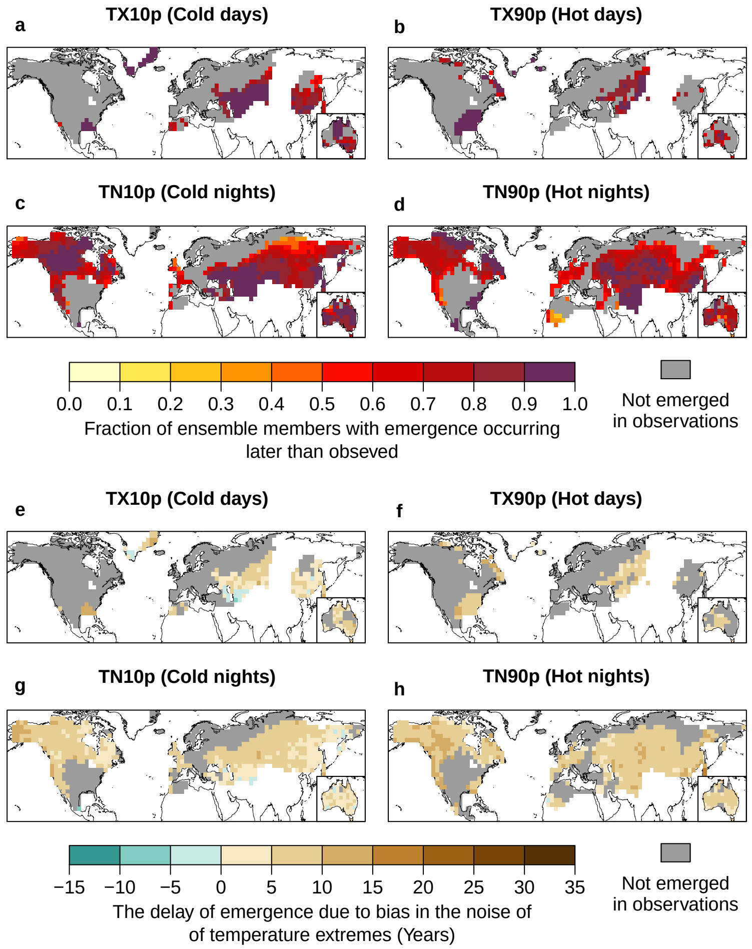

Figure S12: The role of biases in 'noise' in delaying the emergence of persistent changes to TX10p (cold days), TX90p (hot days), TN10p (cold nights) and TN90p (hot nights). The same as Fig S11 but for the results after correcting for bias in the noise. The map is produced using R version 3.0.3 software (https://www.r-project.org/).

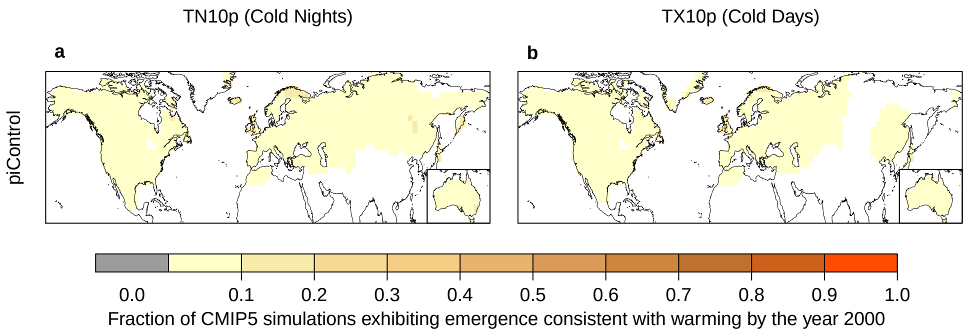

Figure S13: Emergence of persistent changes to TN10p (cold nights) and TX10p (cold days) is unlikely to be explained by internal variability alone. Panels show the fraction of simulations exhibiting emergence consistent with warming by the year 2000 in an ensemble of 540 85-year time series of TN10p (**a**) and TX10p (**b**) drawn from the bias-corrected 'piControl' simulations in terms of a block-bootstrap approach to mimic the length of the 1921-2005 historical period (see Methods). A bias correction is implemented to adjust the simulated internal variability to be consistent in magnitude with the HadEX2 observations. White regions have no data. The map is produced using R version 3.0.3 software (https://www.r-project.org/).

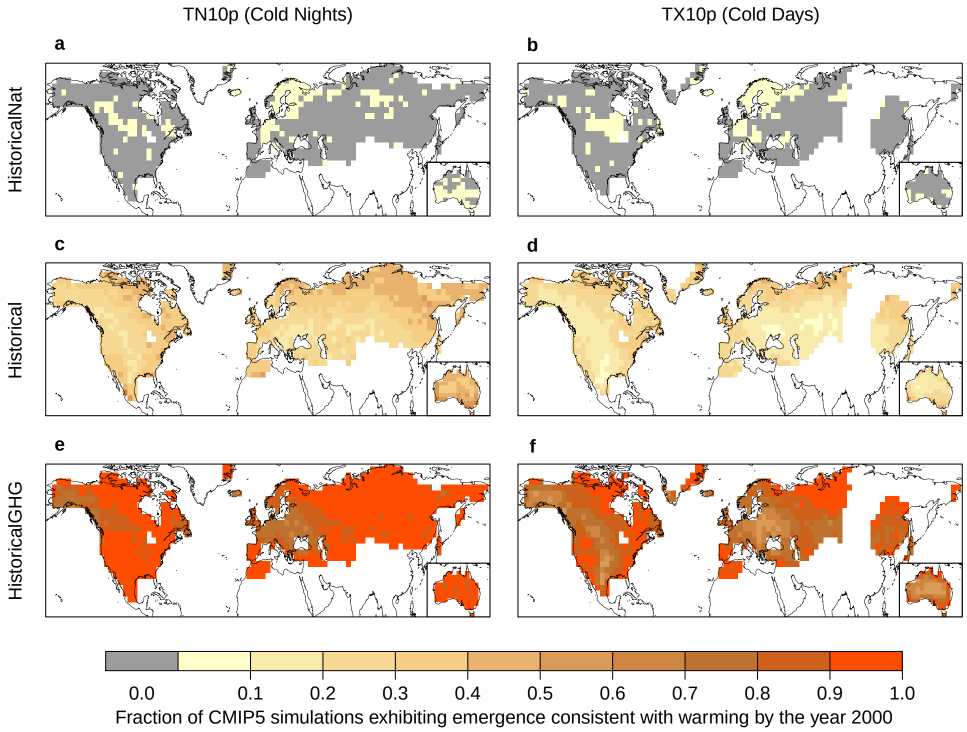

Figure S14: Emergence of persistent changes to TN10p (cold nights) and TX10p (cold days), cannot be explained by natural external forcing, but is likely due to anthropogenic influence, especially anthropogenic emissions greenhouse gases. Panels show the fraction of simulations exhibiting emergence consistent with warming by the year 2000 in 'HistoricalNat' simulations (**a-b**), 'Historical' simulations (**c-d**) and 'HistoricalGHG' simulations (**e-f**) of TN10p (left panels) and TX10p (right panels). White regions have no data. The map is produced using R version 3.0.3 software (https://www.r-project.org/).

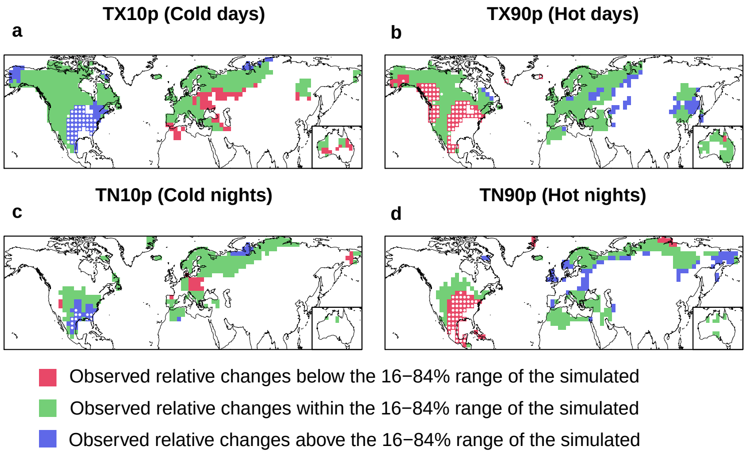

Figure S15: The ratio of the observed 1921-2005 linear trends in TX10p (cold days), TX90p (hot days), TN10p (cold nights) and TN90p (hot nights) to the noise are generally consistent with the 16-84% ranges of the CMIP5 'Historical' simulations over most part of the regions where persistent changes have not occurred yet by the year 2000 in the HadEX2 observations. Panels show whether or not the ratio for TX10p (**a**), TX90p (**b**), TN10p (**c**), and TN90p (**d**) fall below (red), within (green), and above (blue) the 16-84% ranges of simulated by the CMIP5 'Historical' simulations. The map is produced using R version 3.0.3 software (https://www.r-project.org/).
